# MicroRNA‑331 inhibits isoproterenol‑induced expression of profibrotic genes in cardiac myofibroblasts via the TGFβ/smad3 signaling pathway

**DOI:** 10.1038/s41598-021-82226-z

**Published:** 2021-01-28

**Authors:** Fatemeh Yousefi, Bahram M. Soltani, Shahram Rabbani

**Affiliations:** 1 Department of Genetics, Faculty of Biological Sciences, Tarbiat Modares University, P.O. Box 14115-154, Tehran, Iran; 2 Research Center for Advanced Technologies in Cardiovascular Medicine, Cardiovascular Diseases Research Institute, Tehran University of Medical Sciences, Tehran, Iran

**Keywords:** Biological techniques, Cell biology, Computational biology and bioinformatics, Genetics, Molecular biology, Biomarkers, Molecular medicine

## Abstract

Cardiac fibrosis in the failing heart is modulated by activated myofibroblasts, and is a pathology marked by their deposition of extracellular matrix proteins. The TGFβ signaling pathway is important in stimulating fibrosis and therefore seems an attractive new target for anti-fibrotic therapy. The relationship between ncRNAs and TGFβ signaling pathway has been extensively studied. Here, we have provided several lines of evidence to prove that the fibrosis process could be regulated by miR-331 through targeting TGFβ signaling. First, bioinformatics analysis and dual luciferase assay validated a direct interaction between the miR-331 and TGFβ-R1 3′UTR sequence which results in the downregulation of TGFβ signaling pathway. Second, miR-331 expression was inversely related to the expression of a number of genes which are involved in extracellular matrix (ECM) production and deposition processes, both in the in vivo and in vitro fibrosis models. Third, in cultured mouse and human cardiac myofibroblasts (CMyoFbs) under ISO treatment, overexpression of miR-331 decreased the expression level of fibrosis-related genes. Consistently, western blot analysis confirmed that miR-331 overexpression ended in both Smad3 and Col1A1 protein level reduction in mouse cardiac myofibroblasts. Finally, flow cytometry analysis, cyclin D1 and D2 gene expression analysis, and wound-healing assay confirmed the inhibitory effect of miR-331 against cell proliferation and migration in ISO-treated cardiac myofibroblasts. Taken together, accumulative results showed that miR-331 reduced the level of fibrosis-related proteins in cardiac myofibroblasts culture via regulating TGFβ signaling pathway.

## Introduction

Cardiac fibrosis is a pathological force in many cardiac conditions, such as myocardial infarction, hypertension, hypertrophic and dilated cardiomyopathy, which is characterized by a disproportionate accumulation of extracellular matrix (ECM) components in the affected tissue^1–4^. In heart, the accumulation of ECM proteins by activated myofibroblasts changes electric and mechanical properties and contributes to tissue stiffness and diastolic dysfunction of cardiac^5^. Activation of cardiac fibroblasts (CFs) to myofibroblasts is regarded as an important event in the development and progression of cardiac fibrosis^6–8^. Cardiac fibroblasts in the healthy heart provide a balance between new ECM components synthesis and degradation by regulating the action of a large family of cytokines and growth factors, such as Tumor Necrosis Factor-α, Interleukin (IL)-6, IL-10, chemokines, members of the Transforming Growth Factor-β family, and Platelet-Derived Growth Factors^9–11^. However, numerous experimental studies are developing to combat cardiac fibrosis, but molecular mechanisms of cardiac fibroblast‑to‑myofibroblast activation are still unclear.

TGFβ/Smad3 pathway has a pivotal role in cardiac fibrosis by activating fibroblasts to produce ECM in injured heart tissue^11–15^. Upon receptors activation (TGFβR-I and TGFβ-RII), Smad2/3, as intracellular mediators of TGFβ signaling, are phosphorylated and activated, resulting in extracellular matrix protein synthesis induction^16^. Vivar et al.^17^ reported that TGF-β1 induced connective tissue growth factor (CTGF) in cardiac fibrosis, which is a main regulator of extracellular matrix protein. In a rat model with heart pressure overload, it has been proved that myocardial fibrosis could be improved by TGFβ blockade^18^. Moreover, Loss of one TGF-β1 allele in a number of different cardiac fibrosis experimental models altered the development of myocardial fibrosis and diastolic dysfunction resulting in increased survival in 24-month-old mice^19^. So, inquiring the mechanisms of TGFβ signaling pathway in CFs may provide new approaches to treat cardiac fibrosis in cardiovascular diseases.

Noncoding RNAs (ncRNAs) are divided into several classes, including small microRNAs (miRNAs or miRs; ~ 22 nucleotides), long noncoding RNAs (lncRNAs; > 200 nucleotides) and circular noncoding RNAs (circRNAs; > 200 nucleotides and circular)^20^. It has been reported that ncRNAs are involved in the pathogenesis of various cardiovascular diseases, including cardiac fibrosis^21–23^. Accumulating evidence has proved that several miRNAs, such as miR-133, miR-29, miR‑24, miR‑30, miR-26b and miR‑29b, could play a potential anti-fibrotic role in cardiac fibrosis^24–28^. Previous studies showed that TGFβ/Smad3 pathway could mediate cardiac fibrosis via microRNA (miRNA)-dependent mechanisms. For example, Zhang et al.^29^ demonstrated that overexpression of cardiac miR-29b was able to inhibit AngII-induced cardiac fibrosis and improve cardiac dysfunction through the TGFβ/Smad3 pathway. Furthermore, it has been demonstrated that a nuclear hormone receptor, PPARγ, could modulate mRNA expression of TGF-β1 and its targets CTGF and ACTA2 through induction of miR-331-5p in Human Pulmonary Artery Smooth Muscle Cells (HPASMC). The data showed that miR-331-5p had a link to cardiac hypertrophy and exerted its role via repressing PFKP expression as a PPARγ target^30^. Till now, to the best of our knowledge, there is no evidence proven to regulate cardiac fibrosis progression by miR-331.

The present study demonstrated that miR‑331 could protect cardiac myofibroblasts from isoproterenol (ISO)‑induced cardiac fibrosis via the TGFβ/Smad3 signaling pathway. The discoveries may provide a novel understanding of the occurrence of cardiac fibrosis and could be used for the development of a promising therapeutic modality for combating this disease.

## Materials and methods

### Ethical approval of the study protocol

The study protocol was approved by the Animal Experimentation Ethics Committee of Tarbiat Modares University (Tehran, Iran). Experiments were carried out according to the Guidelines for the Care and Use of Laboratory Animals (publication 86-23, revised in 1986; National Institutes of Health (NIH), Bethesda, MD, USA).

### Animal models

Adult C57BL/6 male mice, 25 ± 5 g, were supplied by the Experimental Animal Center of Tehran University of Medical Science (Tehran, Iran) and randomly divided into the cardiac fibrosis model group (n = 30) and the saline group (n = 30). All experiment procedures were performed according to the Guidelines of Animal Experiments from the Ethical Committee for Animal Research of ARRIVE guidelines. The animals were housed individually in cages under hygienic conditions and placed in a controlled environment with a 12 h light–dark cycle at 22 ± 3 °C and 45 ± 10% humidity for 7 days before the experiment. The mice were allowed a commercial standard mouse cube diet and water ad libitum. In the cardiac fibrosis group, Isoproterenol [1-(3,4-dihydroxyphenyl)-2-isopropylamino ethanol hydrochloride] (ISO) was injected subcutaneously once daily for 5 days (20 mg/kg, body wt)^24,31^. To establish the saline group, mice were given equal volumes of saline. The mice were sacrificed by cervical dislocation after deep anesthesia with 2% isoflurane (Baxter International, Inc.) and their hearts were isolated for the subsequent experiments.

### Histological analysis

Mouse hearts were harvested at days 3, 7, 14, 28 after isoproterenol treatment. Tissues were fixed overnight in 10% formalin, followed with paraffin embedding and 4 μm thickness sectioning. To histological examination, they were mounted with coverslips and stained with Masson’s Trichrome. The collagen volume fraction (CVF) was analyzed in five separate views (magnification = original × 400), following the collagen area/total area formula.

### Cardiac fibroblasts (CFs) isolation and activation to myofibroblasts by plating on plastic substrate

Primary mouse cardiac fibroblasts (MCFs) were isolated from Neonatal C57BL/6 mice and human cardiac fibroblasts (HCFs) were obtained from atrial samples obtained as discarded surgical tissue by mechanical tissue enzymatic digestion with collagenases (Roche). Briefly, the tissues were minced and placed in a 1.5 ml-tubes containing collagenase type II and trypsin (ratio, 1:2) then were incubated at 37 °C for 15 min with shaking. Supernatants were then collected in tubes containing growth media (DMEM/LG 10% FBS, 1% penicillin and 1% streptomycin). To collect cells, centrifugation at 800 rpm for 10 min at room temperature (RT) was performed. The cell pellet fraction was then disbursed and plated on 10-cm cell plastic (Biotest Company) culture dishes in growth media for 4 h at 37 °C in humid. In this step, the non-myocytes adhere and the cardiac myocytes (CMs) remain in suspension. The culture plates were washed twice with PBS to detach the weakly attached and non‑adherent cells and then pure CFs were obtained. It is widely accepted that cardiac fibroblasts grown on rigid substrates activate via biomechanical induction to myofibroblasts after plating after early passage^32,33^. In the current study, cells (cardiac myofibroblasts) from the second and third passage were used for experiments and therefore, they are considered as primary myofibroblasts instead of quiescent primary fibroblasts. This notion was supported by molecular evidence.

### Cardiac myofibroblasts treatment

Primary mouse cardiac myofibroblasts and human cardiac myofibroblasts were cultured at a density of 120 × 10^4^ in a 12-well plate for overnight. The next day, cells were incubated with 10 µM Isoproterenol to induce the fibrotic phenotype for 24 and 48 h. Before treatments, cells were transfected with 2000 ng empty pEGFP-CI vector as a negative control (Mock) and or miR-331 constructs by Lipofectamine 2000 (Invitrogen).

### Luciferase reporter assay

The downstream target genes of miR‑331 were predicted using the TargetScan database (https://www.targetscan.org). TGFβ-R1 was recognized as a potential downstream target of miR‑331. Reporter gene construct containing TGFβ-R1 3′UTR was generated using molecular cloning techniques. Briefly, the fragment of the 3′UTR of TGFβ-R1 mRNA that included the binding site for miR-331 was PCR amplified and cloned into the multiple cloning site of the psiCHECK-2 luciferase plasmid (Promega, Fitchburg, WI) downstream from a renilla luciferase gene. The HEK293-T cell line was used for luciferase reporter experiments. Cells were plated 24 h before transfection and treatment in a 48-well plate at a density of 35 × 10^4^ cells/well. Using Lipofectamine 2000 (Invitrogen), cells were transfected with 150 ng of TGFβ-R1 3′UTR or non-target 3′UTR (AXIN1) plasmid, 400 ng of either mock or miR-331 constructs. Transfections were performed in triplicate according to Invitrogen protocol. 48 h after transfection, cells were lysed and luciferase activity was performed by dual-luciferase assays (Promega) and measured by Glomax luminometer (Promega; USA) following the manufacturer’s instructions.

### Wound-healing assay

Mouse cardiac myofibroblasts were seeded in 24-well plates (6 × 10^4^ cells per well), cultured overnight, and transfected. When the culture had reached approximately 90% confluency, the cell layer was scratched by a sterile 20-μl pipette tip. Next, cells were washed 2 times with PBS and cultured with Dulbecco’s modified Eagle's medium (including 10% fetal bovine serum) for up to 48 h. At different time points (0, 24, and 48 h), photographic images of the plates were captured (40 × magnification).

### Flow cytometry

36 h after transfection, cells were harvested and centrifuged at 1200 rpm for 5 min and then washed twice in PBS. Following fixing with 500 μl of 70% cold ethanol for 2 h, the cells were stained with Propidium Iodide (Sigma, USA) and incubated at 37 °C for 30 min away from light. Cells were acquired on a BD FACS Calibur Flow Cytometer (BD Bioscience; USA) and analyzed by Flowing Software version 2.5. The test was performed in triplicates.

### RNA extraction and cDNA synthesis

Total RNA from tissues and CFs was extracted using Trizol Reagent (Invitrogen, USA) following the manufacturer’s protocol and quantified with agarose gel electrophoresis and Nanodrop instrument. cDNA synthesis was performed using 1 µg total RNA, random primer (dN6), oligo(dT)18, and the Prime Script II reverse transcriptase (RT) (Takara, Japan). Poly-A tailing was performed for 1 µg of RNA using 2.5 U Poly A polymerase (NEB, UK) for miRNA detection. A no-RT control was used in parallel to discover any potential nonspecific amplification of contaminated genomic DNA.

### Quantitative real-time PCR

Quantitative RT-PCR was performed using SYBR Green Master Premix Ex Taq Kit (Takara Biotechnology, Korea) in StepOne system (Applied Biosystems, USA). The amplification reaction (20 µl) included 10 µl SYBR green master mix, 0.2 µM of each gene-specific primer and cDNA. The reactions were incubated at 95 °C for 10 min, followed by 40 cycles of 95 °C for 15 s, 60 °C for 60 s and 72 °C for 30 s. All reactions were performed in triplicates. Expression levels were normalized to U6 and GAPDH expression, used as reference genes, following the 2^−ΔΔCt^ formula. The Primers used in this experiment are presented in Table 1.

**Table1:** List of the real time PCR primers.

Gene	Forward primers (5′–3′)	Reverse primers (5′–3′)
GAPDH (Mouse)	CCTGGAGAAACCTGCCAAGTA	GGCATCGAAGGTGGAAGAGT
COL1A1 (Mouse)	ATGGATTCCCGTTCGAGTACG	TCAGCTGGATAGCGACATCG
U6 (Mouse)	GAGAGGGCCAGGGGAGGCATTG	AACTCAAGGTTCTTCCAGTCACG
COL3A1 (Mouse)	GACCAAAAGGTGATGCTGGACAG	CAAGACCTCGTGCTCCAGTTAG
TGF-β1 (Mouse)	ATCCTGTCCAAACTAAGGCTCG	ACCTCTTTAGCATAGTAGTCCGC
SMAD3 (Mouse)	AGTGCATTACCATCCCCAGG	AGGAGGTGGGGTTTCTGGAA
SMAD2 (Mouse & Human)	TGCTCTTCTGGCTCAGTCTG	CTGCCTCCGATATTCTGCTCC
MMP9 (Mouse)	GCGTGTCTGGAGATTCGACT	CTTGGTACTGGAAGATGTCGT
IL-10 (Mouse)	CGGGAAGACAATAACTGCACCC	CGGTTAGCAGTATGTTGTCCAGC
IL-6 (Mouse)	GCAAGAGACTTCCATCCAGTTG	ATAGACAGGTCTGTTGGGAGT
TNF-α (Mouse)	GGTGCCTATGTCTCAGCCTCTT	GCCATAGAACTGATGAGAGGGAG
CCND1 (Mouse)	CAGAGTGATCAAGTGTGACCC	CGTCGGTGGGTGTGCAAGC
CCND2 (Mouse)	AGAACACCCCATGCGTGCTGAG	TGTGTGCCCCTGACCTGGCT
TGFβ-R1 (Mouse)	TGCTCCAAACCACAGAGTAGGC	CCCAGAACACTAAGCCCATTGC
GAPDH(Human)	CCGAGCCACATCGCACAG	GGCAACAATATCCACTTTACCAG
U48 (Human)	TGACCCCAGGTAACTCTGAGTGTGT	AACTCAAGGTTCTTCCAGTCACG
COL1A1(Human)	CGATGGCTGCACGAGTCAC	AACGTCGAAGCCGAATTCC
COL3A1(Human)	GATGGTTGCACGAAACACAC	ATCAGGACCACCAATGTCATAGG
TGF-β1 (Human)	AGTGGACATCAACGGGTTCAC	TGGAGCTGAAGCAATAGTTGGTG
SMAD3(Human)	CTTCCTAAGAGTCAAAGTCCCTGC	CCTGTGCTGGAACATCATCTCAG
MMP9 (Human)	GCCCCAGCGAGAGACTCTAC	CTGGTACAGGTCGAGTACTCC
IL-6 (Human)	GCACTGGCAGAAAACAACCT	CAGGGGTGGTTATTGCATCT
FN1 (Human)	ACAACACCGAGGTGACTGAGAC	GGACACAACGATGCTTCCTGAG
ELN (Human)	GTCTCCCATTTTCCCAGGTG	GATGAGGTCGTGAGTCAGG
TGFβ-R1 3′UTR	CTCGAGTCAGTCAACAGGAAGGCATCAA	GCGGCCGCTTTATCAAACCATCCCTAGCCAAA
AXIN1 3′UTR	CTCGAGAAGGTGGACTGATAGGCTGGT	GCGGCCGCAGAAGACACACCACAGCCAGG
miR-331	CTAGGTATGGTCCCAGGGATCC	AACTCAAGGTTCTTCCAGTCACG

### Western blot

Mouse cardiac myofibroblasts transfected cells were lysed in Ripa buffer following the manufacturer’s protocol (cell signaling) for total protein extraction. Protein concentration was measured by Bradford assay. The extracted proteins were separated by 12% Sodium dodecyl sulfate–polyacrylamide gel electrophoresis (SDS-PAGE) (Sigma, USA), and subsequently transferred to polyvinylidene fluoride (PVDF, Thermo Scientific, USA) membrane. Then, membrane blocking was performed at room temperature for 2 h using 5% BSA (Sigma, USA). The membrane was incubated with the primary antibodies, Smad3, Col1A1 (1/500, Santa Cruz Biotechnology), and GAPDH (1:1000; Santa Cruz Biotechnology), at 4 °C overnight. Following washing, the membranes were incubated with goat anti-mouse IgG-Horse Radish Peroxidase (HRP) (1/3000, Santa Cruz Biotechnology) secondary antibodies for 2 h at room temperature. The bands were visualized using ECL reaction kit (Beyotime, China) and quantified using ImageJ software.

### Statistical analysis

Data are reported as means ± SEM, and sample sizes are mentioned in the figure legends. Unpaired Student’s t-test was used to compare the difference between means. Probability values were considered statistically significant at *p* ≤ 0.05.

## Results

### Experimental evidence for the interaction of miR-331 with TGFβ-R1

**Figure 1:**
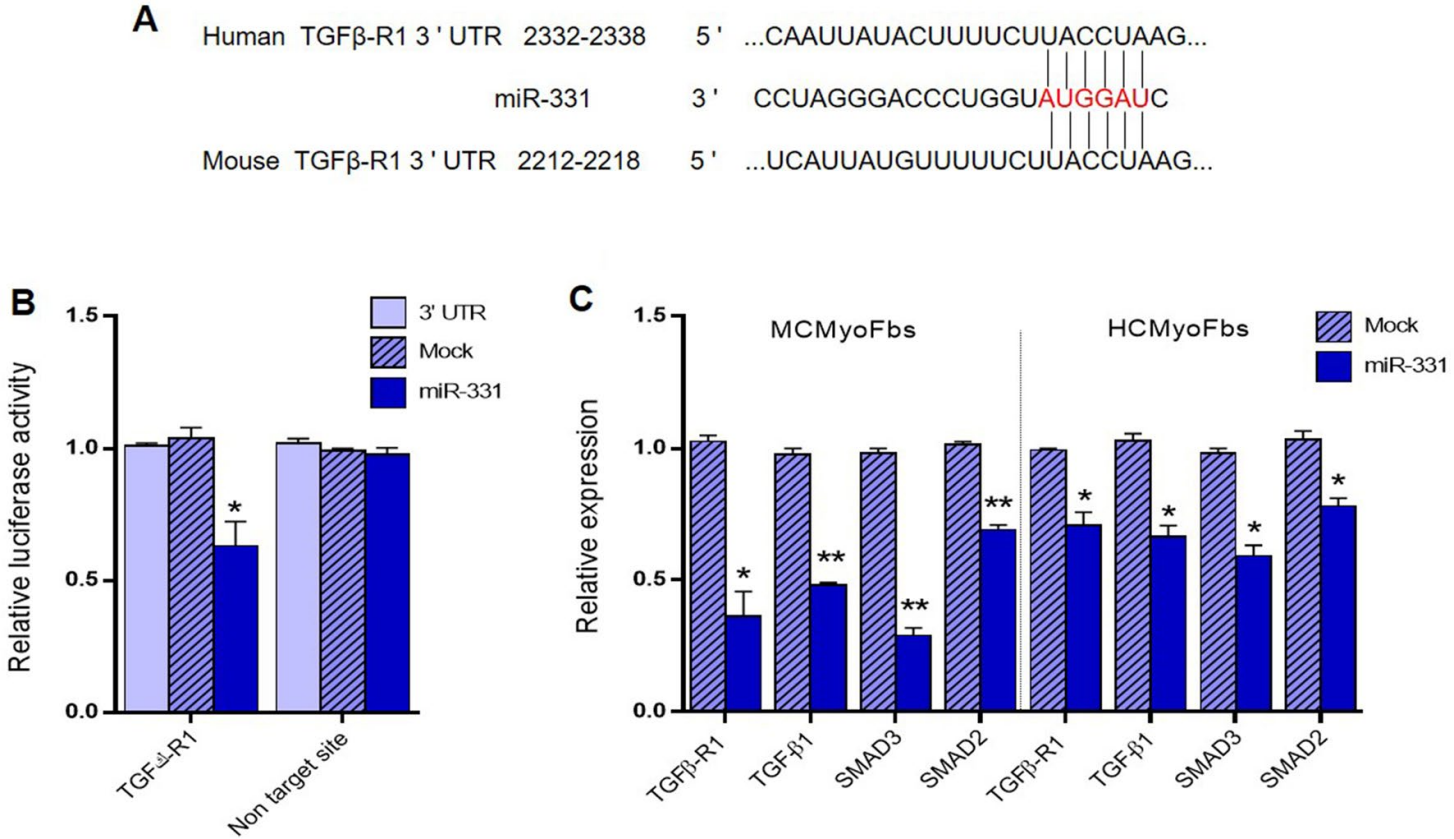
Direct interaction between miR-331 with TGFβ-R1 3′UTR sequence. (**A**) Schematic representation showing a miR‑331 recognition site (MRE) within both mouse and human TGFβ-R1 3′UTRs. (**B**) Shows dual luciferase assay result that supports direct interaction between miR-331 and TGFβ-R1 3′UTR sequence. Co-transfection of HEK293-T cells with miR-331 overexpressing cassette along with the luciferase: TGFβ-R1 3′UTR sequence construct significantly reduced luciferase activity. As a control, when non-target 3′UTR (AXIN1) was used in the assay, made no significant change in the luciferase activity compared with mock transfected cells. (**C**) Shows RT-qPCR results for detection of some genes expression involved in the TGFβ signaling pathway in both MCF and HCF cells under miR-331 overexpression. Downregulation of TGFβ-R1, TGFβ1 and SMAD3 expression was evident in this assay. Assays were performed in triplicate. Means ± SEM was shown. The asterisk mark (*) represents a *p* value < 0.05.

Using a bioinformatics approach through application of Target scan database (www.targetscan.org), miR-331 was predicted to be capable of targeting 3′UTR sequence of TGFβ-R1 mRNA (Fig. 1A). Alignment of the 3′UTR of TGFβ-R1 among human and mouse revealed that the predicted binding sites for miR-331 are highly conserved during evolution, suggesting the potential importance of these binding sites for TGFβ-R1 expression. Subsequently, a dual-luciferase reporter assay was performed to test whether the miR-331 directly interacts with TGFβ-R1 sequence. To this aim, the full-length 3′UTR of the TGFβ-R1 transcript was cloned downstream of luciferase ORF in psiCHECK-2 vector, and was transfected into HEK-293-T cells along with the expression vector ensuring miR-331 overexpression. Reduced luciferase expression at this situation, compared with non-target cloning of the gene (AXIN1 3′UTR) supported direct interaction of miR-331 with 3′UTR sequence of TGFβ-R1 gene (Fig. 1B).

Since TGFβ signaling is known to be involved in cardiac fibrosis^34^, miR-331 effect in this disease was investigated. At first**,** miR-331 was overexpressed both in mouse and human cardiac myofibroblasts which resulted in decreasing expression of three genes involved in TGFβ signaling pathway including, TGFβ-R1, TGF-β1, SMAD2, and SMAD3 (Fig. 1C). This finding pointed to the potential effect of miR-331 in cardiac fibrosis through this pathway.

### Fibrosis induction through isoproterenol treatment of both mouse model and cultured primary cardiac myofibroblasts

**Figure 2:**
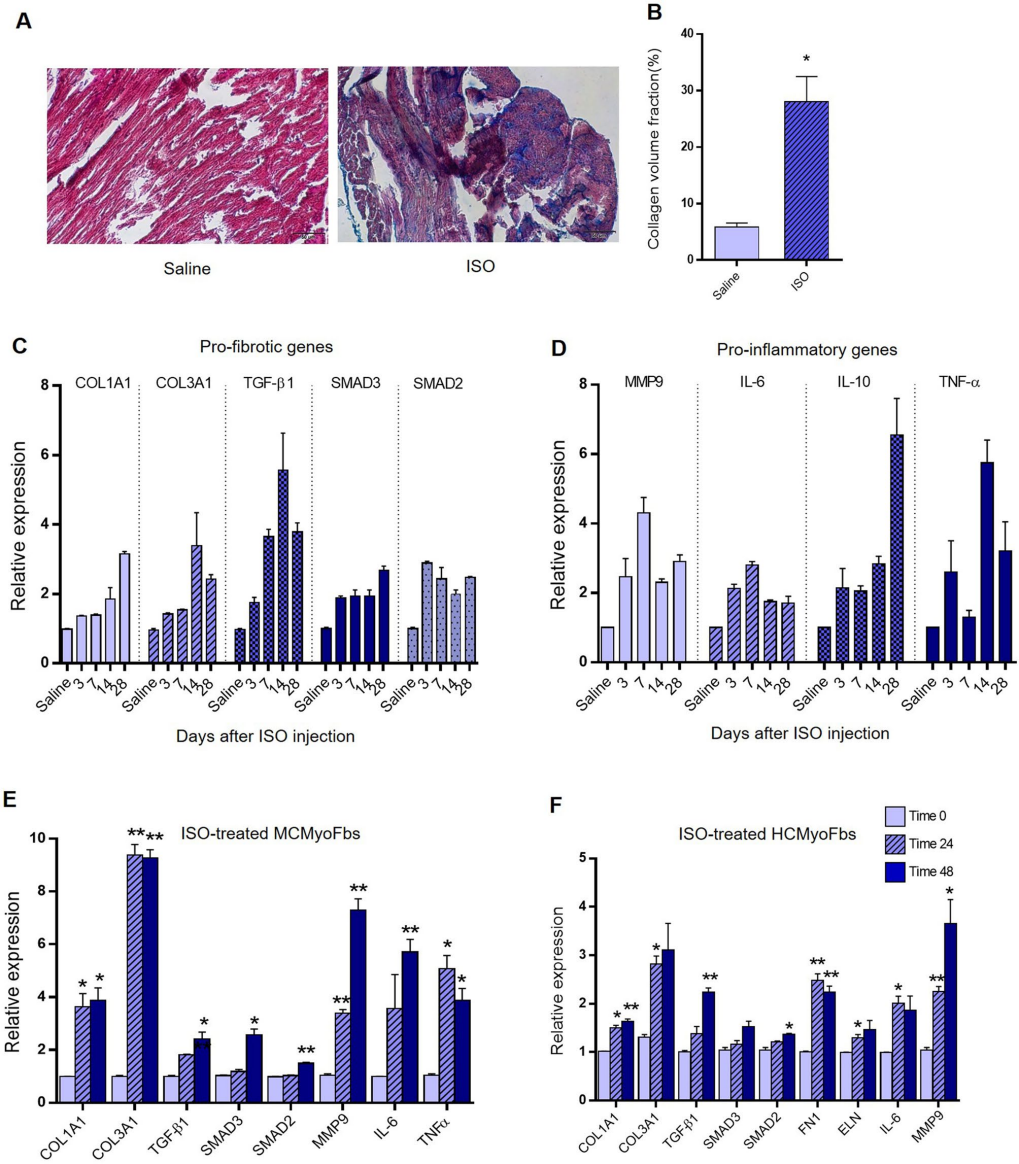
Induction of cardiac fibrosis in mouse model and in vitro condition using Isoproterenol. (**A**) Representative Masson’s Trichrome stain of mouse left heart sections demonstrated fibrotic tissues after ISO injection, compared with the saline group (×400) (n = 3). (**B**) The volume fraction of collagen was markedly increased in ISO treated group of mice, compared with the saline group. Collagen volume was measured using ImageJ software. (**C**,**D**) Shows the upregulation of fibrosis-related genes including, COL1A1, COL3A1, FN1, ELN, TGF-β1, SMAD2, SMAD3, MMP9, IL-10, IL-6, and TNFα at the mRNA levels in the mouse heart at days 3, 7, 14 and 28 following the ISO treatment (each group n = 5). (**E**,**F**) Profibrotic and proinflammatory genes expression were upregulated in both ISO-treated neonatal mouse and human cardiac myofibroblasts (CMyoFbs) after 24 and 48 h. Assays were performed in triplicate. Means ± SEM are shown. The asterisk mark (*) represents a *p* value < 0.05.

In order to explore whether cardiac fibrosis has been occurred, we performed RT‑qPCR and histological staining on heart tissues of treated mouse model. On Masson’s Trichrome staining, heart tissues from isoproterenol-treated mice showed the dynamic change of myocardial structure and increasing collagen fibrils in the outer spaces compared to the saline group (Fig. 2A,B). Furthermore**,** Real-time PCR results showed that the mRNA expression levels of some profibrotic genes (COL1A1, COL3A1, FN1, ELN, TGF-β1, SMAD2, and SMAD3) (Fig. 2C) and proinflammatory genes (MMP9, IL-10, IL-6, and TNFα) (Fig. 2D) have been substantially increased following Isoproterenol treatment in vivo and also in vitro condition (Fig. 2E,F). Our data supported that isoproterenol treatment both in vivo and in vitro has elevated fibrosis in treated mouse heart and cardiac myofibroblasts. Therefore, the fibrosis model has been successful in our experiment.

### Downregulation of miR-331 expression following the in vivo and in vitro fibrosis induction

**Figure 3:**
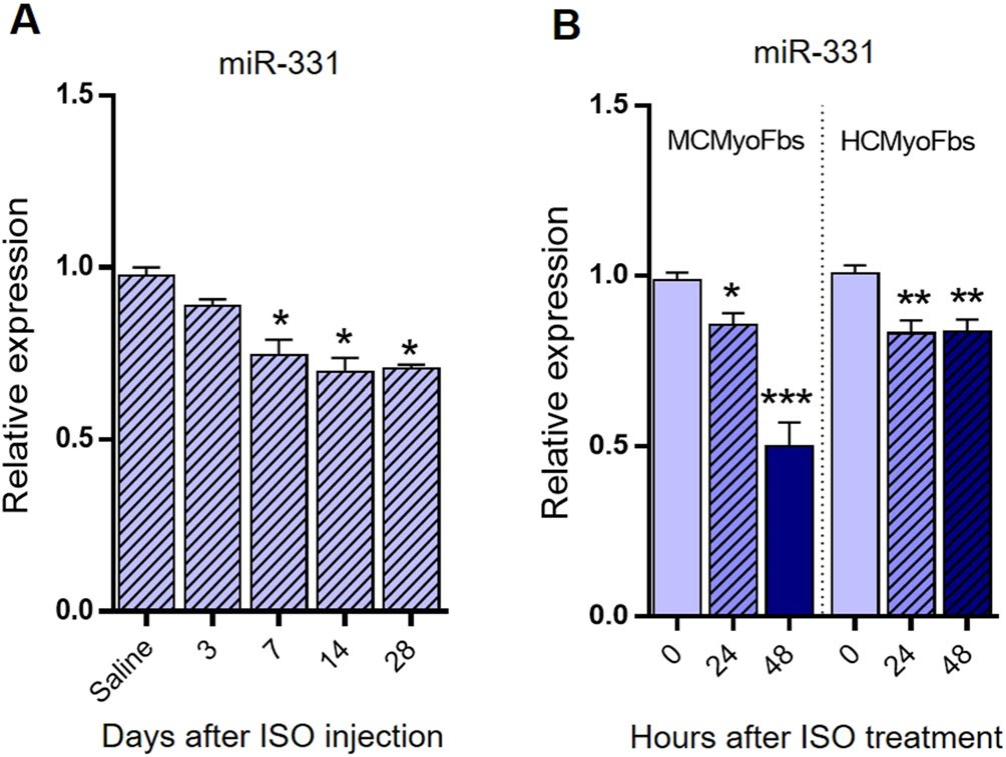
The miR‑331 expression alteration in the cardiac cells of mouse model and cultured cardiac myofibroblasts following ISO‑treatment. (**A**) RT‑qPCR results indicated that miR‑331 level has been reduced in the cardiac fibrosis tissues compared with the saline-treated tissues at days 3, 7, 14 and 28 (each group n = 5). (**B**) The miR‑331-5p relative expression has been downregulated in both ISO‑treated neonatal mouse and human cardiac myofibroblasts compared with the control group after 24 and 48 h. Assays were performed in triplicate. Means ± SEM was shown. The asterisk mark (*) represents a *p* value < 0.05.

The miR‑331 expression level was analyzed in ISO‑treated and saline‑treated mouse cardiac tissues. RT-qPCR results indicated that miR‑331 level has been reduced in ISO‑treated cardiac tissues compared with the saline‑treated group (Fig. 3A). Furthermore, the expression of miR‑331 was analyzed in vitro condition of fibrosis induction in cultured mouse cardiac myofibroblasts and human cardiac myofibroblasts (Fig. 3B). Consistently, RT-qPCR analysis indicated that miR‑331 level has been reduced in ISO‑treated mouse cardiac myofibroblasts and human cardiac myofibroblasts cultured cells, compared with the control groups. Overall, data suggested that miR-331 may act as a regulator in the fibrosis process in heart tissue.

### The miR‑331 overexpression effect on the expression of fibrosis-related genes in cardiac myofibroblasts following fibrosis induction

**Figure 4:**
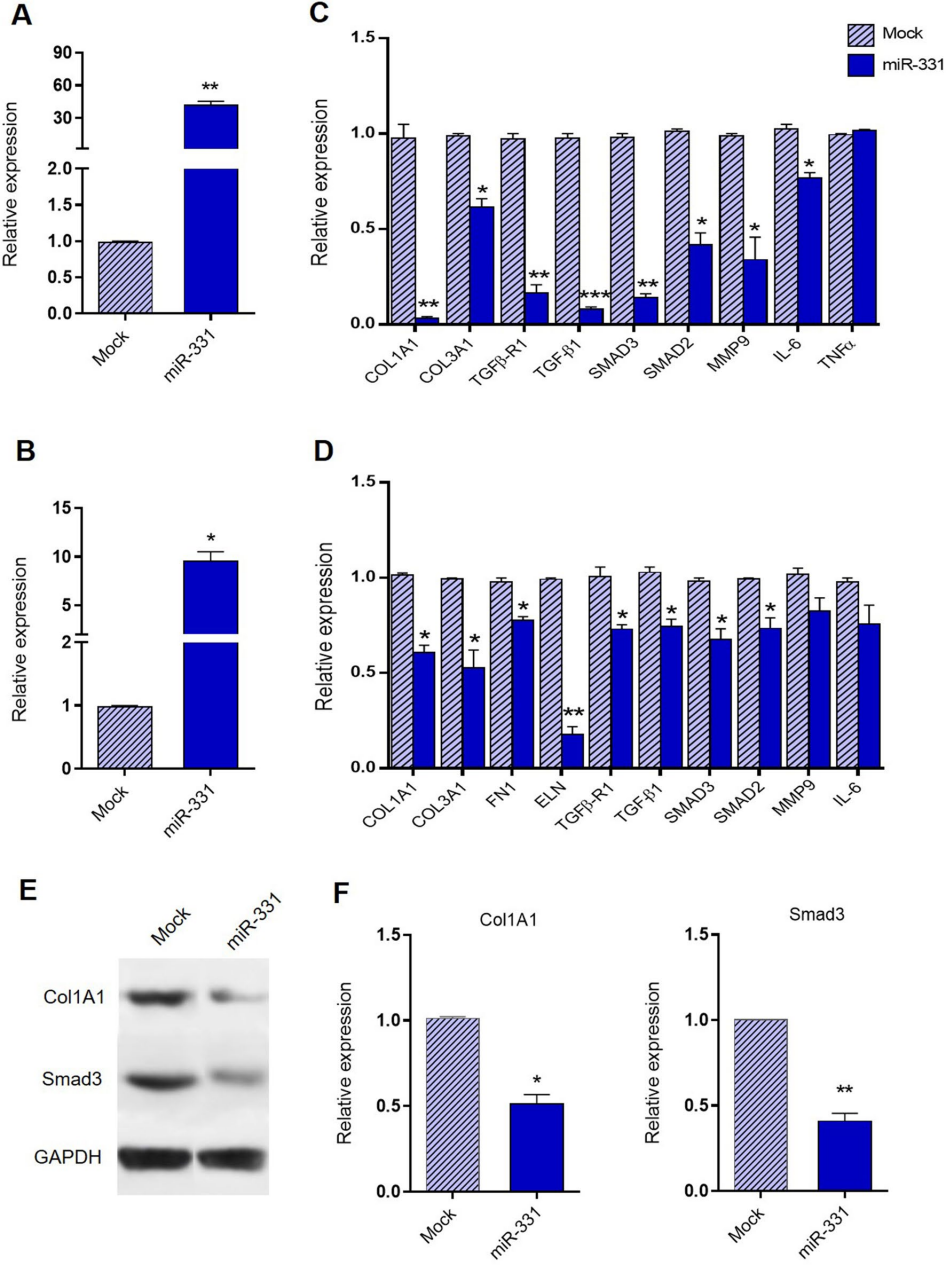
Effect of miR-331 overexpression on some fibrosis-related genes expression in cardiac myofibroblasts following ISO treatment. (**A**,**B**) Shows a successful overexpression of miR-331 both in mouse cardiac myofibroblasts and human cardiac myofibroblasts. (**C**,**D**) Shows the downregulation of fibrosis-related genes including, COL1A1, COL3A1, FN1, ELN, TGF-β1, SMAD2, SMAD3, MMP9, IL-10, IL-6, and TNFα at the mRNA levels in ISO-treated cardiac myofibroblasts under miR-331 overexpression. Assays were performed in triplicate. (**E**,**F**) Overexpression of miR-331 significantly downregulated the level of both Smad3 and Col1A1 proteins, detected by western blot analysis. Protein band quantification was performed using Image-J software in order to ensure the result’s significance. Means ± SEM was shown. The asterisk mark (*) represents a *p* value < 0.05.

The miR-331 was overexpressed in both in mouse and human cardiac myofibroblasts following the ISO treatment and its effect against fibrosis-related genes expression was quantified through RT-qPCR. The overexpression of miR-331 (Fig. 4A,B) was sufficient to reduce the mRNA levels of some profibrotic genes including, COL1A1, COL3A1, FN1, ELN, TGF-β1, SMAD2, and SMAD3 and also proinflammatory genes including, MMP9, IL-6, and TNFα substantially in the cultured treated-cardiac myofibroblasts (Fig. 4C,D). This finding suggested the potential effect of miR-331 in cardiac fibrosis by regulating the fibrosis-related genes expression. Furthermore, western blot analysis was performed in order to confirm the effect of miR-331 on TGFβ1 signaling pathways. miR-331 overexpression in mouse cardiac myofibroblasts significantly downregulated Smad3 and Col1A1 protein levels in comparison to mock transfected cells (Fig. 4E,F).

### The miR‑331 overexpression effect on mouse cardiac myofibroblasts proliferation and migration

**Figure 5:**
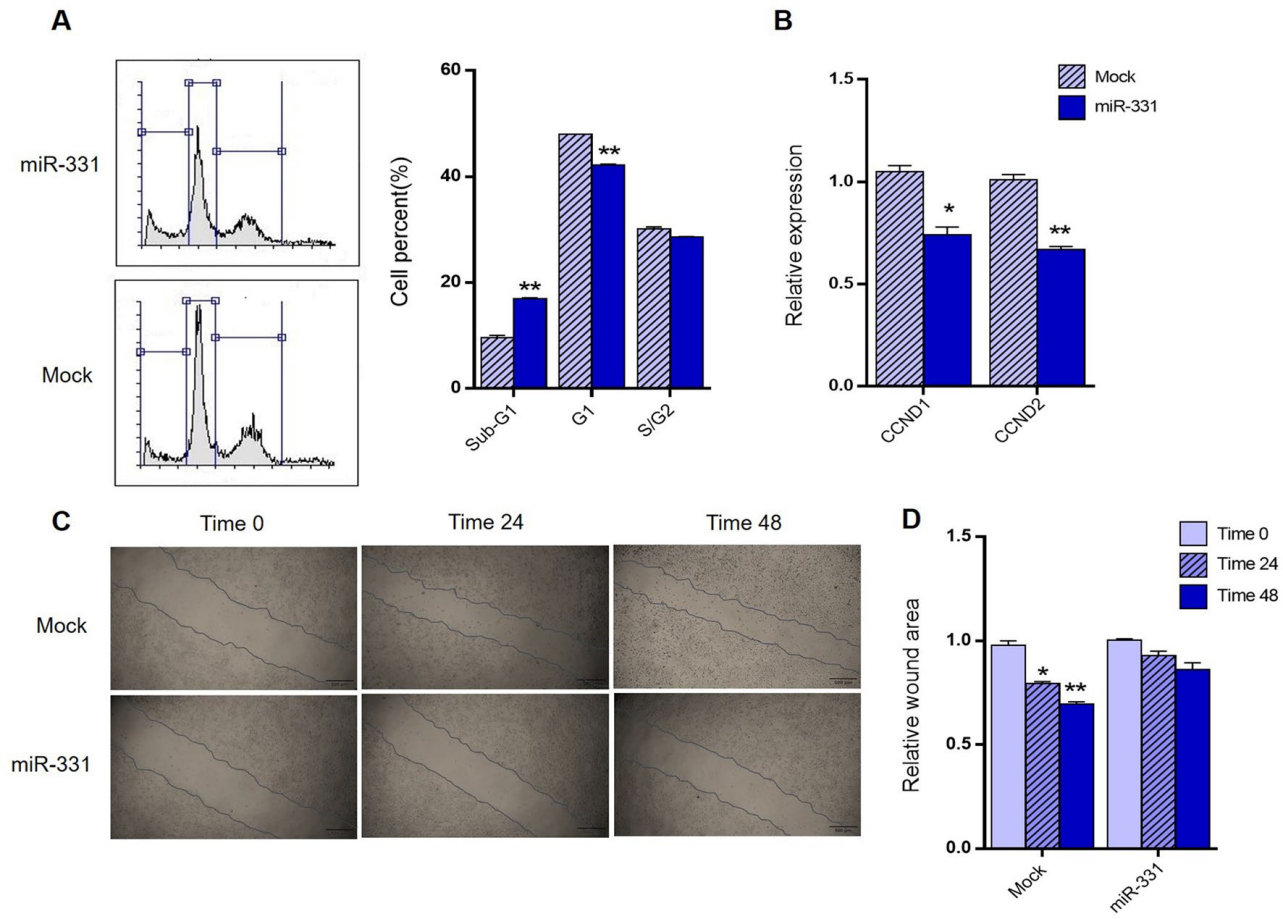
Effects of miR-331overexpression on cell cycle status and cell migration of mouse cardiac myofibroblasts. (**A**) Shows Flow cytometry cell cycle analysis of mouse cardiac myofibroblasts, following the overexpression of miR-331 compared with mock transfected cells. Significantly, sub-G1 cell population has been increased and G1 population fraction has been decreased, following the overexpression of this miRNA. (**B**) RT-qPCR results confirmed that miR-331 overexpression has resulted in significant downregulation of CCND1 and CCND2 genes at the mRNA level compared with mock transfected cells. (**C**) A wound-healing assay in mouse cardiac myofibroblasts transfected with either miR-331 overexpressing cassette or mock vector at 0, 24, and 48 h post-scratching (×40 magnification). (**D**) Quantitative analysis of scratch wound closure revealed miR-331 transfected cells showed no significant cell migration 48 h after of transfection. However, mock transfected cells showed significant cell migration since 24 h and started to progressively increase till 48 h after transfection. Assays were performed in triplicate. Means ± SEM was shown. The asterisk mark (*) represents a *p* value < 0.05.

To investigate the effect of miR-331 on ISO‑treated mouse cardiac myofibroblasts proliferation, precursor of this miRNA was transfected and flow cytometry was performed. Cell cycle analysis revealed that overexpression of miR-331 compared with mock transfected cells, resulted in a considerable increase of the sub-G1 cell proportion and a decrease in the G1 cell proportions (Fig. 5A). Furthermore, to assess miR-331 effect on the expression level of cell cycle-associated genes, cyclin D1 and cyclin D2 (components of the core cell cycle machinery) genes expression was examined. The results indicated that miR-331 overexpression has been followed by decreased mRNA level of cyclin D1 and cyclin D2 in mouse cardiac myofibroblasts, compared to mock transfected cells (Fig. 5B).

Furthermore, a wound healing assay was performed in order to investigate the effect of miR-331 on ISO-treated mouse cardiac myofibroblasts migration. The migration of the ISO-treated cells at different time points (0, 24, 48 h) after scratching was monitored under a microscope (40 × magnification). Although mouse cardiac myofibroblasts cells overexpressing miR-331 showed no significant migration even after 48 h of transfection, the transfection of mouse cardiac myofibroblasts with an empty vector (mock) showed significant migration since 24 h and progressively enhanced till 48 h post-transfection (Fig. 5C,D). Taken together, these results demonstrated that miR-331 expression importantly reduces the migration and suppresses of cell growth in mouse cardiac myofibroblasts.

## Discussion

Cardiac fibrosis commonly occurs in different types of cardiovascular diseases, such as diabetes, ischemic, aging heart disease, which results in heart failure and sudden cardiac death^35–38^. A critical event in cardiac fibrosis is the activation of cardiac fibroblasts (CFs) to myofibroblasts, which includes enhanced level of extracellular matrix (ECM) production and fibrotic processes following cardiac injury^33,39–41^. The alteration of the ECM is the outcome of dysregulation of distinct pro‐fibrotic and anti-fibrotic factors, including cytokines and growth factors, such as Tumor Necrosis Factor-α, Interleukin (IL)-1, IL-10, chemokines, members of the Transforming Growth Factor-β family, IL-11, and Platelet-Derived Growth Factors^42–44^.

Myocardial fibrosis is a major clinical challenge and elaborating its molecular events would help to improve our strategies for increasing the survival rate and the quality of life in patients. Therefore, recognizing the molecular regulators of myofibroblasts activation will increase our ability to treat this problem. Our study demonstrated that miR‑331 could suppress the activation and cell proliferation of ISO‑treated cardiac myofibroblasts through downregulating TGFβ signaling and targeting TGFβ-R1 and potentially other components. It has been reported that some miRNAs including miR-133, miR-30, miR-29 and miR-24 could reduce cardiac fibrosis during the heart failure, by regulating distinct signaling pathway^45–47^. However, some of those, like miR-21 could promote cardiac fibroblast‑to‑myofibroblast transition via downregulating the expression of Jagged1^31^. TGFβ/Smad3 pathway plays a key role in the conversion of fibroblasts to myofibroblasts and also in promoting ECM deposition in the infarct region^48,49^. Previous studies implied that miR-133 and miR-101 could mediate extracellular matrix production through decreasing TGFβ1, TGF-βRII and TGFβ-R1 levels and collagen content in cultured fibroblasts^50–52^. On the other hand, miR-331 plays a central role in the development of some diseases including cardiac disease by controlling critical signaling pathways or downstream gene targets. For example, Zhang et al.^53^ reported that miR‑331-3p led to the dysfunction of Excitation–contraction coupling in heart failure by suppressing Junctophilin 2 expression. The expression of miR-331-3p exhibited a 1.7-fold increase in hypertrophy compared with that in the sham group. However, the expression of miR-331-5p remained unchanged. Here, our preliminary bioinformatics analysis suggested TGFBR1 as a bona fide target gene for the miR-331 (Fig. 1A) and therefore, we intended to investigate its effect on cardiac fibrosis through the involvement of TGFB signaling. Then, dual luciferase assay results verified a direct interaction between the miR‑331 and TGFβ-R1 3′UTR (Fig. 1B). Moreover, overexpression of miR-331 into cardiac myofibroblasts resulted in a significant downregulation of TGFβ-R1, TGF-β1, SMAD2, and SMAD3 mRNA levels which are components of TGFβ signaling pathway (Fig. 1C).

Then using the isoproterenol treatment strategy, cardiac fibrosis was induced in the mouse heart and RT-qPCR results indicated that miR-331 expression level has been reduced in these tissues (Fig. 2A,B). Compared with the saline group, miR-331 expression level has been reduced in all of the tested time points: 3, 7, 14, 28 days after ISO treatment. Also, data showed a negative correlation between miR-331 and some of the profibrotic genes (COL1A1, COL3A1, FN1, ELN, TGF-β1, SMAD2, and SMAD3) expression and also proinflammatory genes (MMP9, IL-6, and TNFα) expression (Figs. 2C,D, 3A). Furthermore, we isolated cardiac fibroblasts from mouse and human tissues and RT-qPCR analysis indicated the activation of fibroblasts to myofibroblast after early passage, consistent to other reports^32,33,54^. Then, fibrosis was induced in these cells by using ISO treatment for 48 h. Consistent to our in vivo results, here again a negative correlation was deduced between the miR-331 and tested fibrosis-related genes expression (Figs. 2E,F, 3B).

Several cytokines including TNF-α, TGFβ, and different interleukins such as IL-10, IL-6, and IL-8 are known to be involved in the development of various inflammatory cardiac diseases^10,55,56^. Increasing the cytokine expression precedes the consequent elevation of local matrix metalloproteinase (MMP) activity (MMP-2 and 9) in the infarct area, as well as the increase in collagen expression^56^. A study by Lijnen et al.^49^ demonstrated that TGF-β1 could induce an increase in collagen production and enhances the abundance of mRNA levels for collagen type I and III in cardiac fibroblasts. Here in the ISO-treated cardiac myofibroblasts, we have shown that overexpression of miR-331, ended in decreased expressions level of, COL1A1, COL3A1, FN1, ELN, TGF-β1, SMAD2, and SMAD3 profibrotic genes. Also, it ended in MMP9, IL-6, and TNFα (proinflammatory markers mRNA level) and Smad3 and Col1A1 (protein level) reduced genes expression, which were accompanied with a decrease in the synthesis of ECM (Fig. 4C–F). The data suggested that miR‑331 exerts a suppressive role on ISO‑induced cardiac fibrosis in cardiac myofibroblasts.

Recent studies have shown miR-331 could inhibit proliferation and promote apoptosis in many cancer diseases. For, example, Guo et al.^57^ had shown that miR-331 directly targeted E2F1 and induced the inhibition of gastric tumor growth. In addition, miR-331-3p can regulate PI3K/Akt and ERK1/2 by targeting HER2 to inhibit development and progression of colorectal cancer^58^. However, the role of miR-331 in regulating CFs proliferation and migration during the fibrosis processes remains blurry and needs to be investigated. In the present study, cell cycle analysis revealed a significant increase at the sub-G1 phase of mouse cardiac myofibroblasts following the miR-331 overexpression (Fig. 5A). It has been reported that cyclin D1 and D2 are two crucial cell cycle regulatory molecules required for the progression of cell proliferation^59,60^. Since a significant modulation was observed at the sub-G1 and G1 phases of the cell cycle after miR-331 overexpression, we checked the transcript level of cyclin D1 and D2 using RT-qPCR. Results confirmed downregulation of cyclin D1 and D2 expression along with miR-331 overexpression in these mouse cardiac myofibroblasts (Fig. 5B). Die et al.^61^ suggested that cyclins, in particular cyclin D1, may cooperate with each other to mediate the cell-promoting effects of TGFβ signaling pathway in aggressive breast cancer cells. Furthermore, wound-healing assay showed that miR-331 overexpression significantly inhibited ISO-treated mouse cardiac myofibroblasts migration after 24 and 48 h of transfection (Fig. 5C,D). These results means that miR-331 reduces the proliferation and migration of mouse cardiac myofibroblasts.

**Figure 6:**
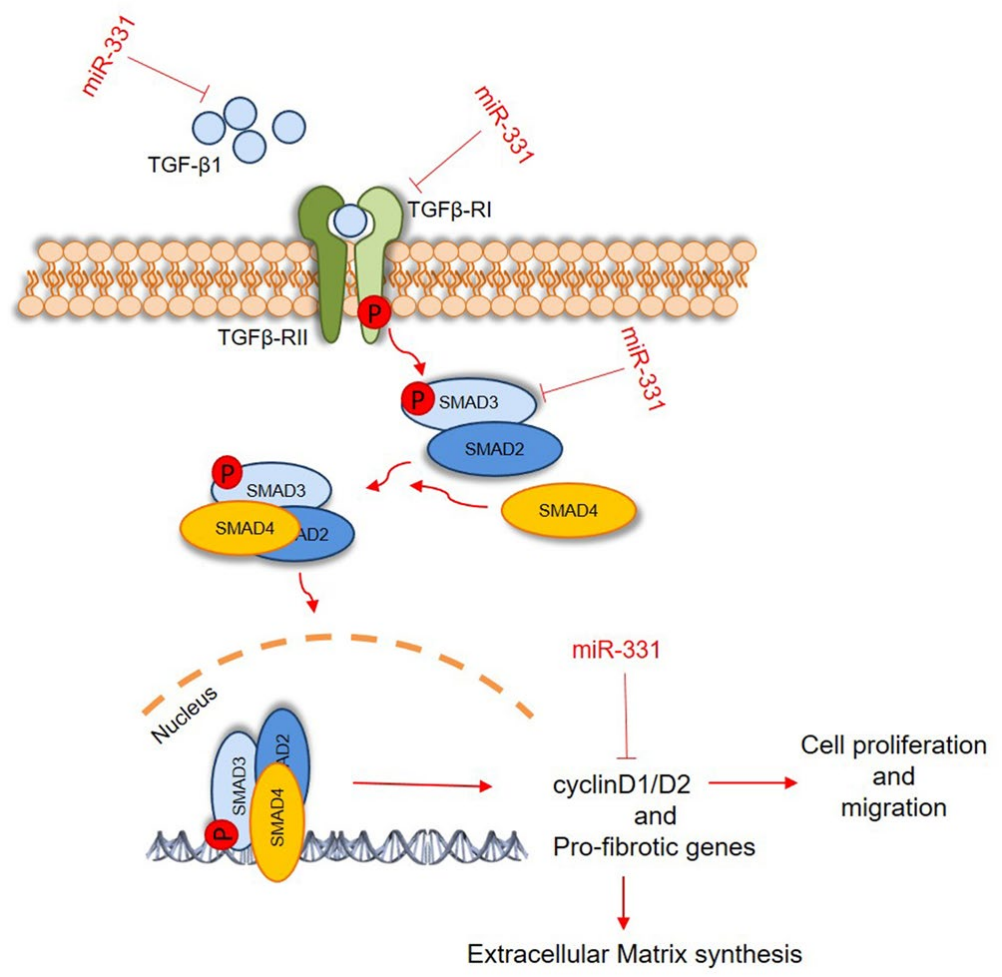
Schematic representation of TGFβ pathway regulation by miR-331. In this model, miR-331 by targeting some genes (TGFβ-R1, TGFβ-1, SMAD2, and SMAD3) results in reduced TGFβ signaling pathway activity which in turn, effects the fibrotic genes expression of cardiac myofibroblasts and other related biological processes, including cell proliferation and migration.

Overall, the present study demonstrated for the first time that miR‑331 could attenuate ISO‑induced cardiac fibrosis through repression of several components of TGFβ signaling in cardiac myofibroblasts culture (Fig. 6). These findings may provide additional evidence for the development of a novel therapeutic strategy for cardiac fibrosis and for understanding the pathogenesis of this disease.

## Supplementary Information

Supplementary Information.

## Abbreviations

ISO: Isoproterenol
TGFβ1: Transforming Growth Factor-β
ncRNA: Non-coding RNA
miR: MicroRNA
COL1A1: Collagen type I alpha 1
COL3A1: Collagen type III alpha 1
FN1: Fibronectin 1
ELN: Elastin
TGFβ-R1: Transforming Growth Factor-β Receptor 1
SMAD3/SMAD2: Mothers against decapentaplegic homolog 3/2
MMP9: Matrix metallopeptidase 9
IL-6: Interleukin 6
IL-10: Interleukin 10
TNFα: Tumor Necrosis Factor-α
MCMyoFbs: Mouse cardiac myofibroblasts
HCMyoFbs: Human cardiac myofibroblasts
ECM: Extracellular matrix
CTGF: Connective tissue growth factor
ACTA2: Actin Alpha 2
LncRNA: Long non-coding RNA
circRNA: Circular RNA
CCND1/2: Cyclin D1/2
PPARγ: Peroxisome Proliferator-Activated Receptor Gamma
PFKP: Phosphofructokinase, platelet
Erk1/2: Extracellular signal-regulated kinase 1/2
PI3K: Phosphoinositide-3-Kinase
3′UTR: 3′Untranslated region
MRS: MicroRNA recognition site
AngII: Angiotensin II
HPASMC: Human Pulmonary Artery Smooth Muscle Cells
n: Number of sample
CVF: Collagen volume fraction
RT-qPCR: Real Time-quantitative Polymerase Chain Reaction
