# Supplementary material for: MicroRNA‑331 inhibits isoproterenol‑induced expression of profibrotic genes in cardiac myofibroblasts via the TGFβ/smad3 signaling pathway
Source: Sci Rep. 2021 Jan 28;11:2548. doi: 10.1038/s41598-021-82226-z (PMC7843612; doi:10.1038/s41598-021-82226-z)
Supplement: Supplementary file 1 — Supplementary Information. [file 41598_2021_82226_MOESM1_ESM.docx]

**SUPPLEMENTARY DATA**

**MicroRNA‑331 inhibits isoproterenol‑induced expression of profibrotic genes in cardiac myofibroblasts via the TGFβ/smad3 signaling pathway**

Fatemeh Yousefi^1^, Bahram M. Soltani^1*^, Shahram Rabbani^2*^

1 Department of Genetics, Faculty of Biological Sciences, Tarbiat Modares University, Tehran, Iran

2 Research Center for Advanced Technologies in Cardiovascular Medicine, Cardiovascular Diseases Research Institute, Tehran University of Medical Sciences, Tehran, Iran

*Corresponding author e-mail: soltanib@modares.ac.ir

*Corresponding author e-mail: sh-rabbani@tums.ac.ir

Tel.: +98-02182884703

Fax: +98-2182884717

P.O. Box: 14115-154

**Supplementary Figure 1**: Western blot analysis of (A) Col1A1, (B) Smad3, and (C) GAPDH protein, following overexpression of miR-331 and C1-Mock in mouse cardiac myofibroblast cells (MCMyoFbs). GAPDH served as loading control. Protein load: 40 µg/lane. Unlabeled lines belong to another study.

**A**


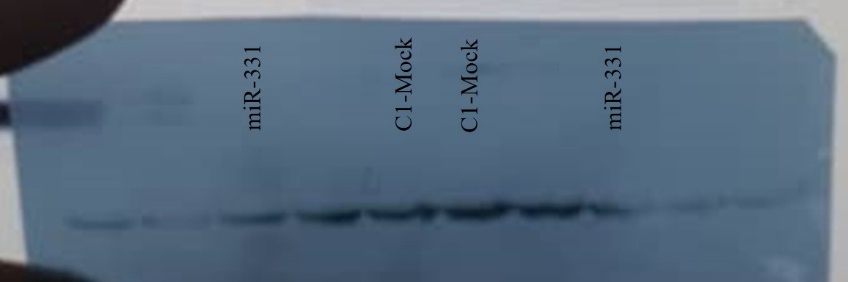


**B**

**
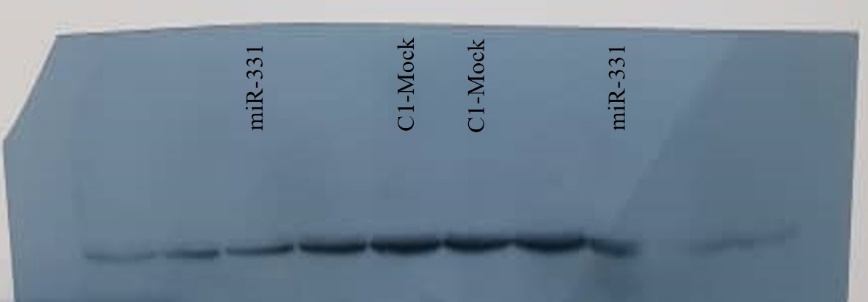
**

**C**

**
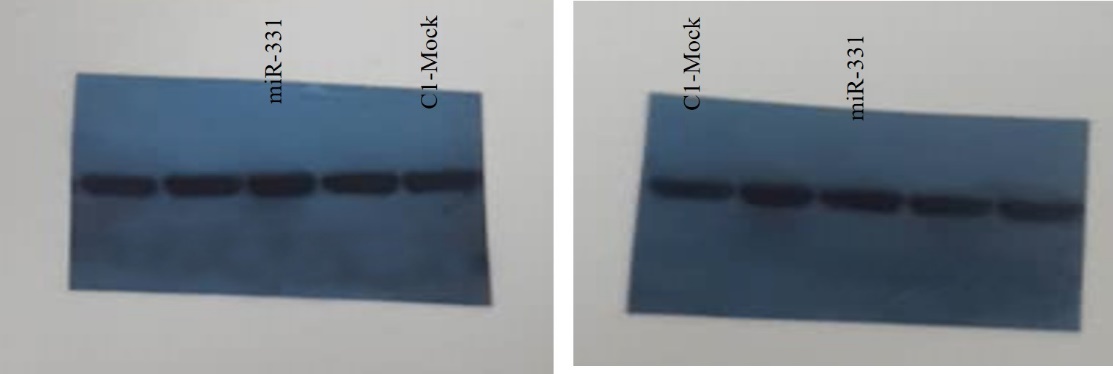
**
